# Targeted Therapies in Pancreatic Cancer: A New Era of Precision Medicine

**DOI:** 10.3390/biomedicines12102175

**Published:** 2024-09-25

**Authors:** Bingyu Li, Qiong Zhang, Claire Castaneda, Shelly Cook

**Affiliations:** 1 University of Wisconsin Hospitals and Clinics, Madison, WI 53792-2460, USA; tianxiaseventh@gmail.com (B.L.); qzhang@uwhealth.org (Q.Z.); ccastaneda@uwhealth.org (C.C.); 2 School of Medicine, Tongji University, Shanghai 200092, China

**Keywords:** pancreatic ductal adenocarcinoma (PDAC), targeted molecular therapies

## Abstract

Pancreatic ductal adenocarcinoma (PDAC), a leading cause of cancer mortality in the United States, presents significant treatment challenges due to its late diagnosis and poor prognosis. Despite advances, the five-year survival rates remain dismally low, with only a fraction of patients eligible for potentially curative surgical interventions. This review aims to comprehensively examine the current landscape of targeted therapies in PDAC, focusing on recent developments in precision medicine approaches. We explore various molecular targets, including *KRAS* mutations, DNA damage repair deficiencies, mismatch repair pathway alterations, and rare genetic fusions. The review discusses emerging therapies, such as PARP inhibitors, immune checkpoint inhibitors, and novel targeted agents, like RET and NTRK inhibitors. We analyze the results of key clinical trials and highlight the potential of these targeted approaches in specific patient subgroups. Recent developments in PDAC research have emphasized precision oncology, facilitated by next-generation sequencing and the identification of genetic and epigenetic alterations. This approach tailors treatments to individual genetic profiles, improving outcomes and reducing side effects. Significant strides have been made in classifying PDAC into various subtypes, enhancing therapeutic precision. The identification of specific mutations in genes like *KRAS*, along with advancements in targeted therapies, including small molecule inhibitors, offers new hope. Furthermore, emerging therapies targeting DNA repair pathways and immunotherapeutic strategies also show promising results. As research evolves, integrating these targeted therapies with conventional treatments might improve survival rates and quality of life for PDAC patients, underscoring the shift towards a more personalized treatment paradigm.

## 1. Introduction

Pancreatic ductal adenocarcinoma (PDAC) is a type of exocrine pancreatic cancer with over 66,000 new cases diagnosed annually in the United States [1]. It was the fourth leading cause of cancer-related death in 2021 and is projected to become the second leading cause by 2030 [2,3]. Despite the development of various treatments, the five-year survival rate is only 10% [3]. Reasons for this include early dissemination, ineffective systemic therapies, and delayed detection. Currently, surgical resection remains the only potentially curative treatment, but only 15–20% of patients are eligible due to the advanced stage of disease at diagnosis [3]. Even with surgery, the prognosis remains grim; the five-year survival rate post-pancreaticoduodenectomy is about 25–30% for node-negative tumors and 10% for node-positive tumors. These statistics underscore the urgent need for new therapeutic approaches [4,5,6].

Recent years have seen significant progress in PDAC research, particularly in the realm of targeted therapies. Advances in genomic sequencing have enabled the rapid identification of specific genetic and epigenetic alterations that make tumor cells distinct from normal cells. This has opened a new window to precision oncology, where treatments are tailored to the genetic profile of a patient’s tumor, potentially reducing side effects and avoiding over treatment [7]. In a recent study involving 18 patients with advanced or metastatic pancreatic cancer, targeted therapies were administered based on specific genomic alterations [8]. Patients with a genomic matching score of 50% or higher experienced significantly improved clinical outcomes. Specifically, the median overall survival (OS) for these patients was 6.8 months, compared to 3.3 months for those with lower matching scores. Similarly, the median progression-free survival (PFS) was 3.9 months versus 1.8 months. These findings underscore the potential efficacy of personalized targeted therapies in treating advanced pancreatic cancer, particularly when a high degree of genomic matching is achieved.

Currently, several ongoing trials (e.g., the NCI-MATCH) are using next-generation sequencing of multiple genes (gene panel tests) to identify molecular abnormalities in the tumors of patients with refractory cancers. These abnormalities may potentially align with molecularly targeted therapies that are either in clinical trials or approved for treating other cancer types. Two such gene panel tests (the Memorial Sloan Kettering Cancer Center Integrated Mutation Profiling of Actionable Cancer Targets [MSK-IMPACT] and the FoundationOne CDx [F1CDx]) are FDA-approved in the United States.

The classification of PDAC into various subtypes has significant clinical implications and serves as a cornerstone of precision oncology [9]. Programs like “Know Your Tumor” have demonstrated that targeted therapies based on molecular profiles can substantially improve overall survival. For instance, one study found that 26% of PDAC profiles had actionable molecular alterations, leading to a notable improvement in survival rates, with a hazard ratio of 0.42 and a highly significant *p*-value of 0.0004 [10]. This highlights the potential benefits of personalized treatment strategies in enhancing patient outcomes.

Further supporting this approach, a large-scale real-world study involving 1856 US patients with pancreatic cancer provided compelling evidence for the efficacy of precision medicine in PDAC [11]. Within this cohort, 46 patients with actionable mutations who received relevant tailored therapies demonstrated significantly longer median survival compared to both those with actionable mutations who did not receive targeted therapies and those without actionable mutations. These real-world data underscore the promise of personalized treatment approaches in PDAC, demonstrating tangible survival benefits when patients are matched with molecularly guided therapies based on their specific tumor profiles.

Genetic aberrations in PDAC typically fall into two categories: activation of oncogenes and inactivation of tumor suppressors. The most common mutations occur in *KRAS*, TP53, CDKN2A, and SMAD4. Genes involved in chromatin stabilization, remodeling, or editing—such as DNA mismatch repair genes (e.g., mutL homolog 1 [MLH1], mutS homolog 2 [MSH2], and mutS homolog 6 [MSH6]) and homologous repair deficiency (HRD) genes (e.g., breast cancer susceptibility—BRCA1/2 and the partner and localizer of BRCA2, PALB2)—also play significant roles and warrant further study.

The field of targeted therapy for PDAC is evolving rapidly, with current strategies focusing on inhibiting the dysregulated activation of oncogenes, interfering with the inactivation of tumor suppressors, and exploiting biological deficiencies in specific genes. This review highlights the recent advances in targeted therapy that aim to enhance treatment efficacy and improve outcomes for patients with PDAC.

This review aims to comprehensively examine the current landscape of targeted therapies in PDAC, focusing on recent developments in precision medicine approaches. We explore various molecular targets, including *KRAS* mutations, DNA damage repair deficiencies, mismatch repair pathway alterations, and rare genetic fusions. The review discusses emerging therapies, such as PARP inhibitors, immune checkpoint inhibitors, and novel targeted agents, like RET and NTRK inhibitors. We analyze the results of key clinical trials and highlight the potential of these targeted approaches in specific patient subgroups. A summary of these targeted therapies is provided in Table 1, while Table 2 presents an overview of the clinical trials referenced throughout the article.

## 2. Target the Dysregulated Activation of Oncogenes *KRAS*

Over 90 percent of PDAC harbor somatic mutations in the *KRAS* gene [12,13,14], making it a central focus in pancreatic cancer research and treatment strategies. These mutations typically occur early in pancreatic carcinogenesis, as evidenced by their presence in non-invasive precursor lesions, such as pancreatic intraepithelial neoplasia (PanIN). Murine models have convincingly demonstrated that oncogenic *KRAS* is crucial for both the formation of these precursors and the initiation and maintenance of invasive pancreatic cancers [15]. The most common mutations occur at codon 12, with subtype allele frequencies of G12D (40%), G12V (34%), G12R (16%), and G12C (1%) [16]. These mutations result in a protein that is locked in an active GTP-bound state, continuously signaling for cell growth and division. This dysregulated signaling contributes to the aggressive nature of PDAC and is associated with poorer overall survival compared to *KRAS* wild-type tumors [17,18,19].

Given that somatic *KRAS* mutations are both common and early events in pancreatic neoplasia, the *KRAS* gene is an attractive target for therapy. A landmark paper published in 2013 by Shokat and colleagues identified the novel *KRAS-G12C* cysteine-containing switch II pocket and demonstrated the first selective inhibition of *KRAS*-G12C [20]. As a result, Sotorasib and Adagrasib are now included in the NCCN (National Comprehensive Cancer Network) guidelines for treating locally advanced and metastatic pancreatic ductal adenocarcinoma (mPDAC) with *KRAS-G12C* mutations. This inclusion reflects a renewed interest in *KRAS*-targeted therapies, which has led to the development of a diverse range of treatments, including small-molecule inhibitors, interfering RNA (siRNA), *KRAS* vaccines, and inhibitors targeting downstream pathways.

The initial clinical investigation of *KRAS-G12C* inhibitors, known as CodeBreaK 100, was a multicenter, phase I/II trial that evaluated the safety and efficacy of Sotorasib (AMG510) in various advanced solid cancers with *KRAS-G12C* mutations [21]. The study included 129 patients previously treated with multiple therapies. Among 12 patients with PDAC, one achieved a confirmed objective response following several doses of Sotorasib, and eight others maintained stable disease, resulting in a disease control rate of 75%. In a subsequent analysis of the CodeBreak 100 trial, Sotorasib was evaluated in an expanded cohort of 38 patients with metastatic pancreatic ductal adenocarcinoma who had previously received systemic therapy [22]. At a median follow-up of 17 months, objective responses were confirmed in eight patients (21%), all of which were partial responses. The disease control rate was 84%. Median progression-free and overall survival were 4–7 months, respectively. The rate of grade ≥3 toxicity rate for Sotorasib was 16%; the most common toxicities were diarrhea and fatigue (5% each). Other grade ≥3 toxicities included abdominal pain, increases in ALT or AST, pleural effusion, and pulmonary embolism. The follow-up CodeBreaK 101 trial investigating Sotorasib in combination with other therapies is currently underway (NCT04185883).

Additionally, preliminary results from the ongoing KRYSTAL-1 study evaluating Adagrasib (MRTX849) in advanced solid tumors with *KRAS-G12C* mutations showed promising outcomes in patients with PDAC. Among the 21 PDAC patients, the objective response rate (ORR) was 33.3% (7/21), with a disease control rate (DCR) of 81% (17/21). The median progression-free survival (PFS) was reported as 5.4 months (95% CI 3.9–8.2), and the median overall survival (OS) was 8 months (95% CI 5.2–11.8) [23].

Since *KRAS-G12C* mutations are rare in PDAC, research is also focusing on inhibitors for more common *KRAS* mutations, such as G12D, G12V, and G12R, as well as pan/all-RAS inhibitors [17]. The *KRAS-G12D* mutation is particularly challenging to target because it lacks a reactive cysteine in the switch II pocket [24]. MRTX1133, a non-covalent *KRAS*-G12D inhibitor developed in 2021, has shown promise in preclinical studies by inhibiting *KRAS* nucleotide exchange and RAF1 binding, reversing early PDAC growth, and positively altering the tumor microenvironment [25,26,27]. Clinical trials are ongoing for MRTX1133 (NCT05737706) and another *KRAS*-G12D inhibitor, RMC-9805 (NCT06040541). Pan-RAS inhibitors are also being explored, though they present toxicity challenges due to off-target effects [28]. Preliminary data from a phase I trial of the pan-*KRAS* inhibitor RMC-6236 in patients with previously treated mPDAC (n  =  22) and non-small-cell lung cancer (NSCLC) (n  =  11) harboring different *KRAS* mutations excluding G12C (NCT05379985), showed an overall response rate of 36% and a disease control rate of 86% [29].

Progress is being made in *KRAS* vaccine development. Peptide-based vaccines, such as ELI-002, are being developed to stimulate tumor-specific immune responses against *KRAS* mutations in pancreatic ductal adenocarcinoma (PDAC). Preliminary data from the trial (NCT04853017) show promising biomarker reductions and induction of polyfunctional *KRAS*-specific T-cell responses.

RNA interference (RNAi) strategies, including small interfering RNA (siRNA) and microRNA (miRNA), face delivery challenges but have shown potential in early-phase studies [30]. Innovative delivery systems, such as exosomes and biodegradable polymers, are being explored to enhance the stability and targeting of siRNA. Ongoing clinical trials, including the phase II PROTRACT trial, are evaluating these approaches in *KRAS*-mutated PDAC and other solid tumors, with some showing promising results in improving progression-free survival and overall survival [31]. Additionally, mRNA vaccines, like mRNA-5671, are also under investigation for *KRAS*-mutated cancers.

## 3. Target the BRCA or PALB2 Mutation Carriers and Other Homologous Recombination Repair Deficiency Alterations

Genomic instability is a hallmark of nearly all human cancers and includes copy number alterations, gene rearrangements, and mutations, which facilitate the clonal expansion of cancer cells [32]. In PDAC, approximately 25% of patients possess somatic or germline mutations in genes related to the DNA damage repair (DDR) and homologous recombination repair (HRR) pathways. These genes include BRCA1, BRCA2, PALB2, ATM, BAP1, BARD1, BLM, BRIP1, CHEK2, FAM175A, FANCA, FANCC, NBN, RAD50, RAD51, RAD51C, and RTEL1.

A recent meta-analysis of six studies involving 21,842 PDAC cases demonstrated that whole-genome/whole-exome sequencing detects a higher proportion of patients with Homologous Recombination Deficiency (HRD) (24–44%) compared to targeted next-generation sequencing (NGS) at the gene level (14.5–16.5%). The prevalence of germline and somatic HRD mutations varies among patients, with BRCA1 mutations present in 0.9% of cases, BRCA2 in 3.5%, PALB2 in 0.2%, ATM in 2.2%, CHEK2 in 0.3%, FANC in 0.5%, RAD51 in 0.0%, and ATR in 0.1% of cases [33].

Recent research has shed light on the importance of copy number variations (CNVs) in HRR pathways and their impact on PDAC prognosis and treatment strategies [34]. A large single-institute cohort study revealed that amplification of HRR- and receptor tyrosine kinase (RTK)-related genes was associated with poor prognosis. The study identified four molecular subtypes based on CNV patterns and HRR proficiency: repair-deficient, proliferation-active, repair-proficient, and repair-enhanced. The repair-deficient subtype showed the most favorable prognosis and may be more responsive to PARP inhibitors, while repair-enhanced and repair-proficient tumors exhibited higher tumor mutation burden (TMB), suggesting potential responsiveness to immunotherapy.

HRD increases cellular sensitivity to therapies that cause DNA damage or inhibit other DNA repair pathways. Poly (ADP-ribose) polymerase (PARP) plays a crucial role in repairing single-strand DNA breaks via base excision [35,36]. Cells with HRD are then forced to rely on the more error-prone non-homologous end joining (NHEJ) for DSB repair. This reliance on NHEJ not only exacerbates disruptions in the DNA sequence, but also undermines genomic stability, ultimately leading to synthetic lethality [37].

The POLO study, a phase III clinical trial, investigated the efficacy of olaparib (PARP inhibitor, PARP-i) as maintenance therapy for patients with BRCA1/2-mutated metastaticPDAC who had previously undergone platinum-based chemotherapy. Of 3315 patients screened, 154 were randomized in a 3:2 ratio, with 92 receiving olaparib. The results showed a significant extension in progression-free survival (PFS) for the olaparib group compared to the placebo group (7.4 months vs. 3.8 months; hazard ratio (HR) = 0.53) [38]. These findings led to the approval of olaparib by the FDA and the European Medicines Agency (EMA) for use as maintenance therapy in pancreatic cancer patients with germline BRCA1/2 pathogenic variants (PV), following platinum-based chemotherapy [39].

Talazoparib, another promising PARP-i, has shown, in vitro, a significantly higher selectivity and efficacy (20- to over 200-fold greater) against tumor cells harboring BRCA1/2 or PTEN mutations compared to earlier PARP inhibitors [40]. A phase I clinical trial of talazoparib in various BRCA1/2-mutated tumors indicated a favorable safety profile and promising antitumor activity [41]. Ongoing phase II trials are underway to evaluate its efficacy in solid tumors (NCT02286687, NCT03565991).

The success of novel target therapies with PARP1 inhibitors in pancreatic cancer has prompted further research into the role of DDR in pancreatic cancer development and progression. This research aims to broaden the patient population that could benefit from those therapies and to identify new targets for other DDR-focused treatments. Several ongoing trials are exploring the use of PARP inhibitors in PDAC patients, both as a monotherapy and in combination with other treatments [42].

In addition to PARP inhibitors, other emerging therapeutic targets in the DDR pathway for PDAC include the Wee1 kinase [43], which regulates the G2/M checkpoint in the cell cycle, and DNA-dependent protein kinase (DNA-PK) [44,45]. Preclinical and early clinical studies suggest that targeting these proteins could be effective either as monotherapies or in combination with existing therapies. Combining these approaches with PARP inhibitors may help overcome resistance and expand therapeutic options for PDAC [46,47].

Patients with HRD in PDAC show increased sensitivity to platinum-based chemotherapies. These agents, such as Oxaliplatin and Cisplatin, primarily exert their cytotoxic effects by causing DNA damage, including the formation of DNA crosslinks (both intra- and interstrand) and DNA strand breaks [48]. PDAC patients with BRCA1/2 mutations show increased sensitivity to these platinum-containing chemotherapies [49]. Consequently, the current National Comprehensive Cancer Network (NCCN) guidelines recommend first-line treatment with platinum-containing chemotherapy for patients with mPDAC who have a germline mutation in homologous recombination repair (HRR) genes, such as BRCA1/2 or PALB2, provided they have a good performance status.

## 4. Target the Mismatch Repair Pathway

DNA mismatch repair (MMR) is a crucial cellular process responsible for correcting errors such as inappropriate nucleotide insertions, deletions, and mismatches that occur during DNA replication [50]. Key genes involved in this process include MutL protein homolog 1 (MLH-1), PMS1 homolog 2 (PMS-2), MutS homolog 2 (MSH-2), and MutS homolog 6 (MSH-6) [51]. Mutations in these MMR genes, whether germline or somatic, can lead to MMR deficiency (dMMR). This deficiency is associated with both sporadic malignancies and hereditary cancer syndromes. Loss of functional MMR leads to microsatellite instability (MSI), characterized by disruptions in protein synthesis from transcriptional frameshift mutations. MSI can generate in neoantigens, which may enhance the effectiveness of immunotherapies in dMMR/MSI-high tumors, providing a potential target for immunotherapeutic strategies [52].

Immune checkpoints play a critical role in protecting against autoimmunity but can also be exploited by cancer cells to evade immune detection. Immune checkpoint blockade (ICB) therapy includes therapeutic monoclonal antibodies to target immune checkpoints, such as programmed cell death 1 (PD-1) and cytotoxic T-lymphocyte-associated antigen 4 (CTLA-4). While ICB has shown promising results in various solid tumor malignancies, its role in PDAC has been limited, with mixed results from initial trials that included both monotherapies and combination therapies using anti-PD-1 (durvalumab) and anti-CTLA-4 (tremelimumab) [53,54].

Pembrolizumab, an anti-PD-1 antibody, is approved for patients with advanced PDAC that exhibit high microsatellite instability (MSI-H), DNA mismatch repair deficiency (dMMR), or a high tumor mutational burden (TMB). A phase Ib/II trial combining pembrolizumab with gemcitabine and nab-paclitaxel in chemotherapy-naïve metastatic patients demonstrated improved efficacy compared to historical data on gemcitabine and nab-paclitaxel alone [55].

In contrast, monotherapy with Ipilimumab, an anti-CTLA-4 antibody, in locally advanced and metastatic PDAC showed no benefit in a clinical trial (NCT00112580). Furthermore, a combination of Ipilimumab with gemcitabine did not demonstrate increased effectiveness compared to gemcitabine alone in a phase Ib trial for advanced PDAC [56]. Notably, a related study combining higher doses of Tremelimumab (10 or 15 mg/kg) with Gemcitabine resulted in longer overall survival (OS) compared to historical data for gemcitabine monotherapy, suggesting that higher doses of immune checkpoint inhibitors (ICIs) might enhance efficacy [57]. Further, combining Ipilimumab with GVAX, a cancer vaccine in patients with previously treated PDAC, showed some clinical benefit: three out of fifteen patients achieved stable disease, and seven out of fifteen showed a decrease in CA19–9 levels, a marker often elevated in pancreatic cancer [58].

These findings highlight the potential of integrating immunotherapies with traditional chemotherapy or emerging treatments, like PARP inhibitors, personalized neoantigen vaccines, adoptive cell transfer, and modulators of the tumor microenvironment (TME), to improve treatment outcomes in pancreatic cancer.

## 5. SMAD4-Targeted Therapy

SMAD4 is a critical signaling molecule in the TGF-β pathway, which regulates cell proliferation, differentiation, and apoptosis. In PDAC, mutations or loss of SMAD4 occur in about 16% to 44% of patients [59]. The absence or alteration of SMAD4 is linked to more aggressive disease progression, increased metastasis, and poorer outcomes [60].

Recent research highlights the potential of metformin, a commonly used diabetes medication, for treating PDAC, particularly in patients with SMAD4 deficiency. Studies suggest that metformin may inhibit PDAC progression and improve survival rates in patients lacking functional SMAD4, while such benefits are not seen in those with normal SMAD4 expression [61]. This differential response indicates that SMAD4 status could act as a biomarker to identify patients who might benefit most from metformin therapy.

These findings warrant further clinical trials to investigate metformin as a targeted therapy for SMAD4-deficient PDAC. Such research could clarify the mechanisms behind metformin’s anticancer effects and pave the way for more personalized treatment approaches in pancreatic cancer, tailoring therapy to specific genetic profiles.

Despite these promising leads, significant research gaps remain. The exact mechanisms by which metformin exerts its anti-cancer effects in SMAD4-deficient PDAC are not fully understood. Moreover, the optimal dosing and treatment regimens for metformin in this context are yet to be determined.

## 6. RET-Fusion-Targeted Therapy

RET (rearranged during transfection) fusions or alterations occur in 0.6–1.3% of PDAC and 1.35% of *KRAS* wild-type PDAC [16]. Selpercatinib, a selective inhibitor targeting RET gene fusions, has shown promise as a treatment for advanced PDAC patients with this genetic alteration. The LIBRETTO-001 basket trial, which included 45 patients with various solid tumors containing RET fusions, reported an objective response rate of 44% and a median response rate of 24.5 months [62]. Notably, in a subset of 11 patients with advanced PDAC, 54% achieved a partial response, and the median duration of response had not been reached at the time of the last follow-up, which averaged 14.9 months. This suggests a potentially durable response to Selpercatinib in PDAC patients. The FDA has granted accelerated, tissue-agnostic approval to Selpercatinib for patients with locally advanced or metastatic solid tumors that have a RET gene fusion who have progressed following prior treatments and lack satisfactory alternative options.

Additionally, the ARROW study, a multi-cohort, open-label, phase I/II trial, assessed the efficacy of Pralsetinib in patients with RET-altered solid tumors, including *KRAS* wild-type PDAC. All four PDAC patients in the study showed positive responses, with one maintaining a continuous complete response for over 33.1 months, highlighting the impressive potential of RET inhibitors for specific genetic subtypes of PDAC.

Ongoing research, including additional clinical trials on Selpercatinib, Pralsetinib, and newer generation inhibitors (EP0031 and KL590586), continues to explore the potential of RET-targeted therapies for various solid tumors, offering hope for patients with these genetic alterations. Future studies should focus on addressing the identified research gaps to maximize the therapeutic potential of RET inhibitors in PDAC treatment.

Despite promising results, significant challenges remain in RET-targeted therapies for PDAC. The rarity of RET fusions makes large-scale trials difficult and limits the broader applicability of findings. Resistance mechanisms to RET inhibitors in PDAC are not fully understood, and strategies to overcome them need development. Identifying suitable patients requires widespread molecular profiling, which is not yet standard practice. While initial responses to RET inhibitors can be dramatic, their long-term efficacy and impact on overall survival in PDAC patients are still unclear. More research, including long-term follow-up studies, is needed to address these issues and optimize the use of RET-targeted therapies in PDAC treatment.

## 7. NTRK-Fusion-Targeted Therapy

The neurotrophic tropomyosin receptor kinase (NTRK) family consists of transmembrane tyrosine kinases essential for neuronal development and has recently emerged as a target in cancer therapy [63]. Although NTRK fusions are rare in PDAC, occurring in less than 0.5% of cases, these fusions present significant therapeutic opportunities [64]. Larotrectinib, a selective inhibitor for targeting NTRK fusions, received accelerated FDA approval in November 2018 for the treatment of advanced solid tumors with these genetic abnormalities. This approval was based on a basket study that demonstrated significant efficacy across various tumor types with NTRK fusions [65].

Following Larotrectinib, Entrectinib, an oral inhibitor targeting TRL, ROS1, and ALK, also gained FDA approval in 2019. This approval was for treating advanced solid tumors with aberrations in NTRK1/2/3, ROS1, or ALK, supported by data from the STARTRK-1 (NCT02097810), ALKA-372-001 (NCT02097810), and STARTRK-2 (NCT02568267). The ongoing STARTRK-2 study continues to enroll patients, including those with PDAC. Preliminary data presented at the ASCO 2022 annual meeting indicated the inclusion of four PDAC patients [66]. Updated year 2020 guidelines from ASCO endorse use of Larotrectinib or Entrectinib for second-line therapy for individuals with TRK fusion-positive pancreatic cancer [67].

Despite promising results, significant challenges remain in NTRK-targeted therapies for PDAC. The rarity of NTRK fusions in PDAC (occurring in less than 0.5% of cases) limits patient eligibility and complicates large-scale trials and resistance studies. Identifying suitable patients requires comprehensive molecular profiling, which is not yet standard practice and poses cost and accessibility issues. While initial responses to NTRK inhibitors can be dramatic, their long-term efficacy and impact on overall survival in PDAC patients need further investigation. These challenges highlight the need for ongoing research to improve patient identification, develop strategies to overcome resistance, and assess the sustained efficacy of these therapies.

## 8. Discussion

Advancements in next-generation sequencing (NGS) and bioinformatics have dramatically transformed the landscape of pancreatic ductal adenocarcinoma (PDAC) research. These technologies have facilitated the discovery of driver mutations and aberrant pathways, leading to the identification of novel therapeutic targets. Extensively utilized in clinical trials, NGS and bioinformatics enable detailed genomic and transcriptional profiling. The goal is to identify predictive biomarkers that guide therapy decisions and uncover new, actionable targets.

According to ASCO guidelines, early testing for actionable genomic alterations is recommended for patients post-first-line therapy. This includes both germline and tumor (somatic) testing, targeting abnormalities like microsatellite instability/mismatch repair deficiency, BRCA mutations, and NTRK gene fusions. The results from these tests can lead directly to targeted therapies, such as PARP inhibitors, PD-1 checkpoint inhibitors, TRK fusion inhibitors, and facilitate enrollment in clinical trials [67]. In this article, we included the current molecular target therapy/clinical trials, and they demonstrated significant improvement compared to traditional therapy.

Despite recent advances in targeted therapies for PDAC, several significant challenges remain. The complex tumor microenvironment, characterized by dense stroma and immunosuppressive features, continues to impede drug delivery and efficacy [68,69]. Tumor heterogeneity and clonal evolution contribute to treatment resistance, while the limited availability of tumor tissue for comprehensive molecular profiling hinders personalized treatment approaches [70]. Moreover, the rapid development of resistance mechanisms necessitates continuous monitoring and adaptation of treatment strategies [71]. The high cost of precision medicine approaches and the need to balance these with improved patient outcomes present additional hurdles in clinical implementation.

Future directions in PDAC targeted therapy research should focus on developing more effective drug combinations that simultaneously target multiple pathways [72]. Improving drug delivery methods to overcome the dense stroma is crucial, as is exploring novel immunotherapy approaches tailored to the unique immune microenvironment of PDAC [73]. The potential of liquid biopsies for non-invasive molecular profiling and treatment monitoring should be further investigated. Conducting larger, biomarker-driven clinical trials is essential to validate precision medicine approaches in PDAC. Additionally, addressing the marked cachexia and metabolic derangement associated with PDAC could significantly improve patient outcomes and treatment efficacy. Future research should also aim to eradicate PDAC stem cells to prevent recurrence and develop strategies to reduce the peritumoral stroma that supports tumor growth and impedes therapeutic access.

The integration of targeted therapies with standard treatment protocols, including chemotherapy and radiation, offers a robust approach to managing PDAC. Clinical trials that combine agents like immune checkpoint inhibitors with vaccines or chemotherapy are showing promise, suggesting potential synergistic effects that could significantly enhance patient outcomes. These trials suggest potential synergistic effects that could significantly enhance patient outcomes by improving both survival rates and quality of life.

## 9. Conclusions

While genomic sequencing and targeted molecular therapy hold the promise of substantial benefits for patients with PDAC, the challenges remain still. These include the high cost of these advanced therapies and the need for widespread access to specialized skills and technologies. Nonetheless, as the field continues to evolve, the potential for personalized treatment strategies to revolutionize PDAC care becomes increasingly apparent. This review highlights the significant improvements brought about by targeted therapies in comparison to traditional treatment modalities, offering a hopeful prospect for future PDAC management.

## Figures and Tables

**Table 1:** Targeted therapies in pancreatic ductal adenocarcinoma (PDAC).

Targeted Therapy	Target	Mechanism	Description
*KRAS* Inhibitors	*KRAS* Mutations	*KRAS* inhibitors target the *KRAS* protein, which is involved in cell signaling pathways that control cell growth and division. By inhibiting *KRAS*, these drugs aim to interrupt the proliferation of cancer cells.	*KRAS* mutations, particularly G12C, G12D, G12V, and G12R, are common in PDAC. Sotorasib and Adagrasib are KRAS inhibitors showing promise in clinical trials.
PARP Inhibitors	BRCA1/2 Mutations	PARP inhibitors block the PARP enzyme, crucial for repairing single-strand DNA breaks. In cancer cells with BRCA mutations, this leads to accumulation of DNA damage and cell death.	Olaparib is used for PDAC patients with BRCA1/2 mutations, especially after platinum-based chemotherapy. Other PARP inhibitors, like talazoparib, are being studied.
Immune Checkpoint Inhibitors	PD-1/PD-L1	These inhibitors block the interaction between PD-1 on immune cells and PD-L1 on cancer cells, enhancing the immune system’s ability to attack cancer cells.	Pembrolizumab is approved for PDAC with high microsatellite instability (MSI-H) or mismatch repair deficiency (dMMR). Combining immunotherapy with chemotherapy, vaccines, or other agents may enhance efficacy.
SMAD4-Targeted Therapy	SMAD4 Mutations	SMAD4 is part of the TGF-β signaling pathway, which regulates cell growth and apoptosis. Targeting SMAD4 aims to restore normal signaling and inhibit tumor progression.	Metformin has shown potential in treating PDAC with SMAD4 deficiency, possibly by affecting cancer cell metabolism and growth
RET Inhibitors	RET Fusions	RET inhibitors block the activity of RET proteins, which can drive cancer cell growth when abnormally activated.	Selpercatinib targets RET gene fusions, showing durable responses in PDAC patients.
NTRK Inhibitors	NTRK Fusions	NTRK inhibitors block the activity of TRK proteins, which can be abnormally activated in some cancers, leading to uncontrolled cell growth.	Larotrectinib and Entrectinib are approved for treating tumors with NTRK fusions, including PDAC.

Abbreviations: PDAC: pancreatic ductal adenocarcinoma; PARP: poly (ADP-ribose) polymerase; PD-1: programmed cell death protein 1; PD-L1: programmed death ligand 1; RET: rearranged during transfection; NTRK: neurotrophic tropomyosin receptor kinase; TRK: tropomyosin receptor kinase; TGF-β: transforming growth factor beta.

**Table 2:** Clinical trials related to targeted therapies in PDAC.

NCT Number	Phase	Therapy	Target	Patient Population	Outcomes/Expected Results	Publication Status/Expected Results
NCT03600883	I/II	Sotorasib (AMG510)	*KRAS* G12C	Advanced solid tumors with *KRAS* G12C mutation	In 12 PDAC patients: 1 PR, 8 SD (75% DCR)	Published
NCT04185883	I/II	Sotorasib + other therapies	*KRAS* G12C	Advanced solid tumors with *KRAS* G12C mutation	Ongoing	Estimated primary completion date: April 2024
NCT03785249	I/II	Adagrasib (MRTX849)	*KRAS* G12C	Advanced solid tumors with *KRAS* G12C mutation	In 21 PDAC patients: 33% ORR, 81% DCR, 5.4 mo mPFS	Published
NCT05737706	I	MRTX1133	*KRAS* G12D	Advanced solid tumors with *KRAS* G12D mutation	Ongoing	Estimated primary completion date: December 2024
NCT06040541	I	RMC-9805	*KRAS* G12D	Advanced solid tumors with *KRAS* G12D mutation	Ongoing	Estimated primary completion date: June 2025
NCT05379985	I	RMC-6236 (pan-*KRAS* inhibitor)	*KRAS* (excluding G12C)	Previously treated mPDAC and NSCLC with *KRAS* mutations	36% ORR, 86% DCR	Preliminary data presented at ASCO 2023; full publication pending
NCT04853017	I/II	ELI-002 (*KRAS* peptide vaccine)	*KRAS* G12D, G12V, G12R	*KRAS*-mutated solid tumors	Ongoing; promising biomarker and immune response data	Estimated primary completion date: December 2025
NCT04015622	II	siRNA + chemotherapy	*KRAS*	Locally advanced PDAC	Improved PFS and OS	No published results found; trial is ongoing with an estimated primary completion date of December 2024
NCT02184195	III	Olaparib vs. placebo (maintenance)	BRCA1/2	Germline BRCA-mutated mPDAC after platinum chemo	Improved mPFS with olaparib (7.4 vs. 3.8 mo, HR 0.53)	Published
NCT02286687 NCT03565991	II	Talazoparib	BRCA1/2, PTEN	Advanced solid tumors with BRCA1/2 or PTEN mutations	Ongoing	NCT02286687: Completed, no results posted; estimated study completion date was December 2020. NCT03565991: Recruiting; estimated primary completion date is December 2025
NCT00112580	II	Ipilimumab + allogeneic tumor cell vaccine	Immune checkpoint	Previously treated PDAC	3/15 SD, 7/15 decreased CA19–9	Published
NCT04185883	I	Adavosertib (AZD1775) + gemcitabine + radiation	Wee1 kinase	Locally advanced PDAC	Ongoing; prior data show tolerability and activity	Estimated primary completion date: April 2024
NCT03157128	I/II	Selpercatinib	RET fusion	RET fusion-positive solid tumors	In 11 PDAC patients: 54% ORR, durable responses	Published
NCT03037385	I/II	Pralsetinib	RET fusion	RET fusion-positive solid tumors including PDAC	4/4 PDAC patients responded (1 CR > 33 mo)	Published
NCT02576431 NCT02122913	I/II	Larotrectinib Entrectinib	NTRK fusion	NTRK fusion-positive solid tumors	FDA-approved for NTRK+ solid tumors, including PDAC	Published

Abbreviations: NCT, ClinicalTrials.gov identifier; PDAC, pancreatic ductal adenocarcinoma; PR, partial response; SD, stable disease; DCR, disease control rate; ORR, objective response rate; mPFS, median progression-free survival; mo, months; mPDAC, metastatic PDAC; PFS, progression-free survival; OS, overall survival; HR, hazard ratio; CR, complete response; FDA, US Food and Drug Administration.
